# CARMN orchestrates angiogenesis from behind the opera scenes: signing love letters to the endothelium

**DOI:** 10.1172/JCI197708

**Published:** 2025-10-15

**Authors:** Shivangi Pande, George Ishak, Fahimeh Varzideh, Gaetano Santulli

**Affiliations:** 1 Department of Medicine, Wilf Family Cardiovascular Research Institute, Einstein-Mount Sinai Diabetes Research Center (ES-DRC), Fleischer Institute for Diabetes and Metabolism (FIDAM), Albert Einstein College of Medicine, New York, New York, USA.; 2 Department of Molecular Pharmacology, Institute for Aging Research, Institute for Neuroimmunology and Inflammation (INI), Albert Einstein College of Medicine, New York, New York, USA.

## Abstract

Chronic limb-threatening ischemia (CLTI), the advanced stage of peripheral artery disease (PAD), remains a leading cause of morbidity and limb loss. Effective vascular regeneration strategies will require increased understanding of molecular mechanisms underlying angiogenesis. Recent evidence revealed a new role for the vascular smooth muscle cell–enriched (VSMC-enriched) long noncoding RNA (lncRNA) CARMN in endothelial angiogenesis and postischemic vascular repair. CARMN was downregulated in both human CLTI muscle tissue and murine ischemia models. In VSMCs, CARMN deficiency suppressed a specific miRNA-mediated paracrine signaling axis that regulates Hedgehog signaling. In mice, deleting CARMN caused impariment in capillary growth and blood flow recovery after limb ischemia, an effect that was reversed by restoring miR-143-3p or silencing the Hedgehog inhibitor HHIP. The identification of lncRNA-mediated crosstalk between VSMCs and endothelial cells in PAD pathophysiology reveals possible therapeutic targets for CLTI and underscores the translational potential of RNA-based strategies in ischemic vascular disease.

In the landscape of peripheral artery disease (PAD), particularly its advanced stage known as chronic limb-threatening ischemia (CLTI), therapeutic innovation has long been hampered by a limited understanding of the cellular crosstalk that governs vascular repair (1). CLTI remains a major cause of morbidity, often culminating in limb loss (2). Despite efforts to develop therapies based on angiogenic growth factors, clinical translation has fallen short (3). A persistent limitation has been the underappreciation of intercellular communication pathways, particularly the interactions between vascular smooth muscle cells (VSMCs) and endothelial cells (ECs) that regulate capillary growth and vascular regeneration in ischemic tissue (4, 5). In the current issue of *The Journal of Clinical Investigation*, Zhai, Jamaiyar, and collaborators (6) addressed this gap with both elegance and rigor. Their study advances this field by identifying a paracrine signaling mechanism wherein a smooth muscle cell–enriched long noncoding RNA (lncRNA), CARMN (cardiac mesoderm enhancer-associated noncoding RNA) (7, 8), regulates endothelial angiogenic activity through a miRNA-mediated Hedgehog signaling axis.

## Paracrine regulation links VSMCs to ECs

CARMN was previously known for its role in the VSMC phenotypic modulation that is observed in in atherosclerosis (9). In the present work, Zhai, Jamaiyar, and colleagues demonstrated that it was markedly downregulated in the gastrocnemius muscle of patients with CLTI as well as in mice following hindlimb ischemia. Strikingly, CARMN-knockout (CARMN-KO) mice displayed impaired blood flow recovery and reduced capillary density, correlating with elevated tissue necrosis (6). These findings underscore CARMN as a critical determinant of vascular regeneration. A key strength of the study lies in its exploration of the VSMC-to-EC signaling axis. Although CARMN is not expressed in ECs, the authors discovered that supernatants from CARMN-deficient VSMCs suppressed EC proliferation, sprouting, and network formation.

**Figure 1:**
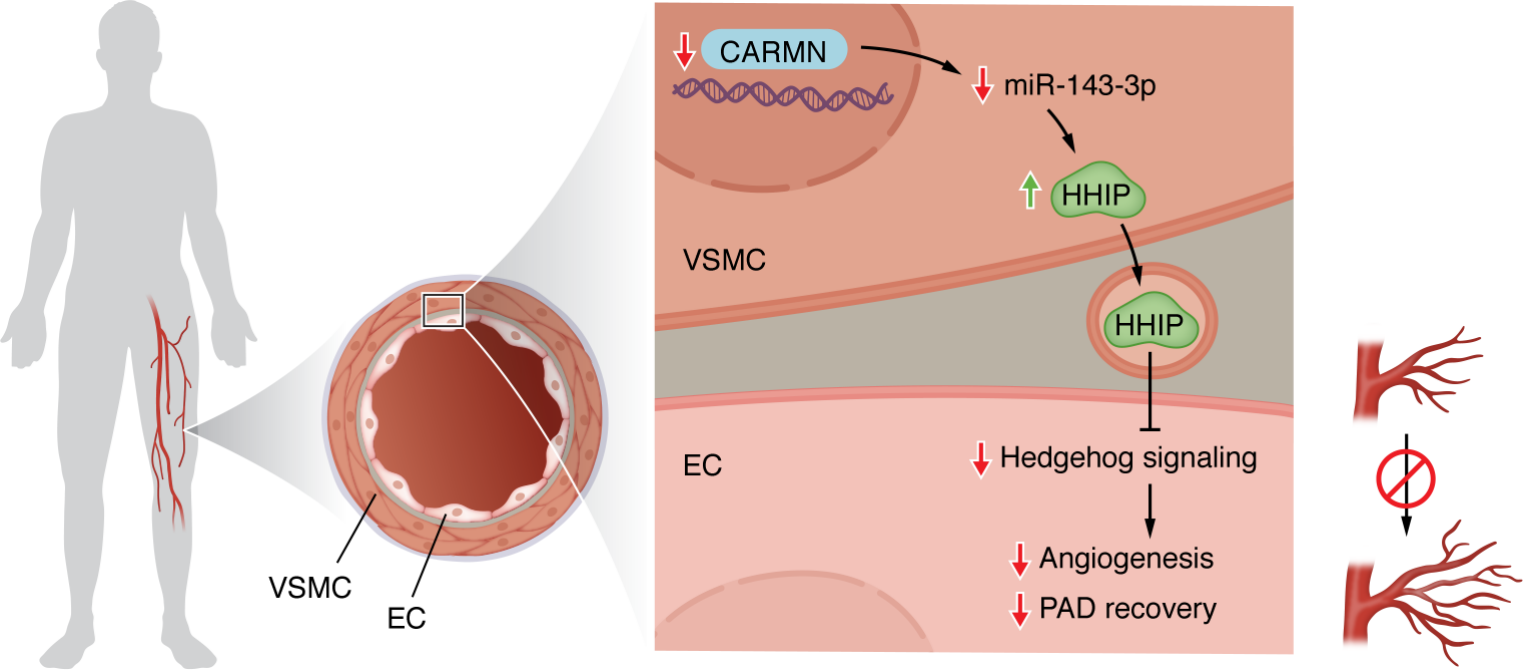
Intercellular paracrine cross talk between smooth muscle and endothelium. Through a mechanism that involves the suppression of miR-143-3p levels, CARMN deficiency in vascular smooth muscle cells (VSMCs) triggers the upregulation of Hedgehog-interacting protein (HHIP), an antagonist of Hedgehog signaling. HHIP then impairs proliferation and network formation of endothelial cell (EC), revealing a functional coupling between VSMC gene regulation and EC angiogenic potential.

This paracrine effect reveals a functional coupling between VSMC gene regulation and EC angiogenic potential. Through RNA sequencing and pathway enrichment analyses, the authors identified downregulation of Hedgehog signaling in CARMN-deficient models. Specifically, they highlighted an increase in the expression of Hedgehog-interacting protein (HHIP), a known antagonist of Hedgehog signaling (10, 11). Mechanistically, CARMN regulated the abundance of HHIP by modulating levels of miR-143-3p, a microRNA that directly targets HHIP (Figure 1). They also performed a particularly compelling set of rescue experiments: reconstitution of miR-143-3p or silencing of HHIP in CARMN-KO mice restored endothelial angiogenic capacity and improved blood flow in ischemic limbs (6). These data not only validated the identification of a miR-143-3p/HHIP/Hedgehog signaling axis in vascular repair but also provide translational relevance by suggesting miR-143-3p as a potential therapeutic candidate.

## Bridging a knowledge gap in PAD pathophysiology

The present results are well substantiated by molecular data, histological analysis, and functional perfusion assays. The study is indeed distinguished by its comprehensive experimental design, including human transcriptomic datasets, murine ischemia models, in vitro angiogenesis assays, and multiple layers of genetic and pharmacological manipulation (6). While earlier investigations had linked CARMN to VSMC differentiation and vascular disease progression (7, 12, 13), Zhai, Jamaiyar, and colleagues have revealed a previously undescribed role for intercellular communication in EC regulation, expanding the functional landscape of lncRNAs in vascular biology (14, 15).

While the study is undoubtedly robust, certain limitations merit discussion. For instance, the mechanisms regulating CARMN expression under ischemic stress remain to be fully elucidated. Understanding whether hypoxia-inducible factors or other stress-responsive elements modulate CARMN transcription could offer further therapeutic entry points. Moreover, although the paracrine effects of CARMN-deficient VSMCs on ECs were well demonstrated, the complete profile (and modalities) of secreted factors mediated by CARMN remains largely undefined. Proteomic and metabolomic analyses could uncover additional CARMN-dependent mediators contributing to endothelial dysfunction.

Lastly, the systemic delivery of miR-143-3p mimics or HHIP inhibitors in humans will require careful consideration of biodistribution, off-target effects, and delivery efficiency (16, 17).

## Clinical implications

CARMN and its downstream effectors represent promising therapeutic targets in CLTI, particularly for patients deemed unsuitable for revascularization procedures (18). Given the challenges of delivering growth factors or stem cells in ischemic tissue (19, 20), targeting endogenous regulatory pathways via noncoding RNAs could offer a more precise and durable strategy (21, 22). Furthermore, CARMN levels in patient muscle biopsies or circulating EVs could serve as a biomarker for ischemic severity or angiogenic potential, aiding in risk stratification and treatment planning (23). In summary, the findings of Zhai, Jamaiyar, and colleagues open new frontiers in our understanding of vascular regeneration in limb ischemia. By unveiling the CARMN/miR-143-3p/HHIP axis as a key modulator of angiogenesis, they provide a compelling case for targeting lncRNA-mediated pathways in PAD. As we move toward a new era of RNA-based therapeutics (24, 25), this work exemplifies how decoding noncoding RNA biology can illuminate novel treatment strategies for complex vascular diseases.

## Version 1 10/15/2025

Electronic publication
